# Supplementary material for: Conformational changes in the essential E. coli septal cell wall synthesis complex suggest an activation mechanism
Source: Nat Commun. 2023 Jul 31;14:4585. doi: 10.1038/s41467-023-39921-4 (PMC10390529; doi:10.1038/s41467-023-39921-4)
Supplement: Supplementary file 3 — Description of Additional Supplementary Files [file 41467_2023_39921_MOESM3_ESM.pdf]

Description of Additional Supplementary Files:

Supplementary Data 1. Spreadsheet summarizing the frequency of all persistent (>10% of the last 500 ns of simulation) hydrogen bonds formed at important interfaces in the complex. Each sheet corresponds to a different interface, and hydrogen bond frequencies are listed for each system simulated.

Supplementary Data 2. Directory containing coordinates for predicted structures and conformers after 1 microsecond of MD simulation, both for the protein and for both protein and membrane.

Supplementary Movie 1. Epifluorescence time-lapse imaging of single Halo-FtsB molecule in M9-glucose, corresponding to Figure 1A (top). Scale bar, 0.5 µm.

Supplementary Movie 2. Epifluorescence time-lapse imaging of single Halo-FtsB molecule in M9-glucose, corresponding to Figure 1A (bottom). Scale bar, 0.5 µm.

Supplementary Movie 3. One-microsecond simulation of the FtsQLBWI complex.

Supplementary Movie 4. One-microsecond simulation of FtsWI embedded in a lipid bilayer. Phosphorus (dark purple) and oxygen (light purple) atoms of the polar head groups are shown.

Supplementary Movie 5. One microsecond simulation of the FtsQLBWI complex with bound activator FtsN.
