# Supplementary material for: Conformational changes in the essential E. coli septal cell wall synthesis complex suggest an activation mechanism
Source: Nat Commun. 2023 Jul 31;14:4585. doi: 10.1038/s41467-023-39921-4 (PMC10390529; doi:10.1038/s41467-023-39921-4)
Supplement: Supplementary file 11 — Reporting Summary [file 41467_2023_39921_MOESM11_ESM.pdf]

nature portfolio

Corresponding author(s): Jie Xiao, Zach Hensel

Last updated by author(s): Jun 20, 2023

Reporting Summary

Nature Portfolio wishes to improve the reproducibility of the work that we publish. This form provides structure for consistency and transparency in reporting. For further information on Nature Portfolio policies, see our Editorial Policies and the Editorial Policy Checklist.

Statistics

For all statistical analyses, confirm that the following items are present in the figure legend, table legend, main text, or Methods section.

n/a	Confirmed	
☐	☒	The exact sample size (*n*) for each experimental group/condition, given as a discrete number and unit of measurement
☐	☒	A statement on whether measurements were taken from distinct samples or whether the same sample was measured repeatedly
☒	☐	The statistical test(s) used AND whether they are one- or two-sided<br>*Only common tests should be described solely by name; describe more complex techniques in the Methods section.*
☒	☐	A description of all covariates tested
☐	☒	A description of any assumptions or corrections, such as tests of normality and adjustment for multiple comparisons
☐	☒	A full description of the statistical parameters including central tendency (e.g. means) or other basic estimates (e.g. regression coefficient) AND variation (e.g. standard deviation) or associated estimates of uncertainty (e.g. confidence intervals)
☒	☐	For null hypothesis testing, the test statistic (e.g. *F*, *t*, *r*) with confidence intervals, effect sizes, degrees of freedom and *P* value noted<br>*Give P values as exact values whenever suitable.*
☒	☐	For Bayesian analysis, information on the choice of priors and Markov chain Monte Carlo settings
☒	☐	For hierarchical and complex designs, identification of the appropriate level for tests and full reporting of outcomes
☒	☐	Estimates of effect sizes (e.g. Cohen's *d*, Pearson's *r*), indicating how they were calculated

Our web collection on statistics for biologists contains articles on many of the points above.

Software and code

Policy information about availability of computer code

Data collection	Custom journals were written in Metamorph 7.8.13.0 software to acquire single-molecule images.<br>Molecular dynamics simulation package NAMD (2.14) was used for generating trajectory data.
Data analysis	ImageJ software (1.53f51) and the ThunderSTORM plug-in (1.3-2014-11-08)were used to identify the fluorophore localization for single-molecule tracking analysis. Code for analyzing single-molecule tracking data is available from the Xiao Laboratory GitHub repository (XiaoLabJHU; https://github.com/XiaoLabJHU/SMT_Unwrapping).<br>Python libraries MDAnalysis (1.0.0) and dynetan (1.0.1) were used for analyzing molecular dynamics data.

For manuscripts utilizing custom algorithms or software that are central to the research but not yet described in published literature, software must be made available to editors and reviewers. We strongly encourage code deposition in a community repository (e.g. GitHub). See the Nature Portfolio guidelines for submitting code & software for further information.

Data

Policy information about availability of data

All manuscripts must include a data availability statement. This statement should provide the following information, where applicable:

- Accession codes, unique identifiers, or web links for publicly available datasets
- A description of any restrictions on data availability
- For clinical datasets or third party data, please ensure that the statement adheres to our policy

The plasmids and E. coli strains used in this study are available from the corresponding authors upon request. Code for analyzing single-molecule tracking data is available from the Xiao Laboratory GitHub repository (XiaoLabJHU). Full all-atom trajectories will be made available in the Anton 2 database also upon publication. Files with coordinates for predicted structures and for structures following 1-µs MD are included as supplementary data.

Research involving human participants, their data, or biological material

Policy information about studies with human participants or human data. See also policy information about sex, gender (identity/presentation), and sexual orientation and race, ethnicity and racism.

Reporting on sex and gender	N/A
Reporting on race, ethnicity, or other socially relevant groupings	N/A
Population characteristics	N/A
Recruitment	N/A
Ethics oversight	N/A

Note that full information on the approval of the study protocol must also be provided in the manuscript.

Field-specific reporting

Please select the one below that is the best fit for your research. If you are not sure, read the appropriate sections before making your selection.

☒ Life sciences ☐ Behavioural & social sciences ☐ Ecological, evolutionary & environmental sciences

For a reference copy of the document with all sections, see nature.com/documents/nr-reporting-summary-flat.pdf

Life sciences study design

All studies must disclose on these points even when the disclosure is negative.

Sample size	No specific sample size calculation or statistic method was made. The sample size varied in different experiments described below and in the manuscript.<br>For single-molecule tracking experiments, at least 100 trajectories (N reported in figure legends) were monitored to reconstruct the distribution and cumulative probability density function for fitting. The errors of mean were clearly listed in the main text and figure legends. The single-molecule movement speeds showed a good normal distribution, which means the sample size is sufficient.<br>For phenotype and fluorescent imaging experiments, at least 100 cells from 2 independent replicates, were chosen except in cases where the phenotype prevented large sample sizes due to lethal phenotype. This was done to ensure the standard error of the mean was small enough to compare average cell lengths.
Data exclusions	Fluorescence spots with an intensity over 3 sigma of the peak intensity were removed from analysis since they likely include more than one molecule. Short trajectories (shorter than 5 time points) of a single molecule are excluded because they cannot be accurately classify or quantify to moving or free diffusion molecules based on our simulation.
Replication	The replicates varied in different experiments described below and in the manuscript.<br>For single-molecule tracking experiments five to ten biological replicates were done by culturing different source colonies of E. coli from the same genotype. The cell growth, drug treatment, and imaging experiments were completed at different days with other conditions such as temperature or cell density kept as similar as possible. All attempts at replication were successful.<br>For phenotype and fluorescent imaging experiments at least two biological were done by culturing different source colonies of E. coli from the same genotype. The cell growth and imaging experiments were completed at different days with other conditions such as temperature or cell density kept as similar as possible. All attempts at replication were successful.

Randomization	Samples were not allocated to groups.
Blinding	The blinding varied in different experiments described below and in the manuscript.<br>The single-molecule tracking experiments, the data collection is intrinsically blinded since the information from single molecules was randomly collected and not be able to choose with bias. Data analysis was not blinded considering the analysis pipeline is more computer-based and standard.<br>The phenotype and fluorescent imaging experiments were not blinded due to the clear visible differences in the phenotypes. All data analysis was performed the same across all samples to ensure equal treatment.

Reporting for specific materials, systems and methods

We require information from authors about some types of materials, experimental systems and methods used in many studies. Here, indicate whether each material, system or method listed is relevant to your study. If you are not sure if a list item applies to your research, read the appropriate section before selecting a response.

Materials & experimental systems

n/a	Involved in the study	
☐	☒	Antibodies
☒	☐	Eukaryotic cell lines
☒	☐	Palaeontology and archaeology
☒	☐	Animals and other organisms
☒	☐	Clinical data
☒	☐	Dual use research of concern
☒	☐	Plants

Methods

n/a	Involved in the study	
☒	☐	ChIP-seq
☒	☐	Flow cytometry
☒	☐	MRI-based neuroimaging

Antibodies

Antibodies used	Primary antibody:polycolonal rabbit anti-FtsI sera (1:50 000 dilution; From: Dr. Savid S. Weiss). Secondary antibody: HRP goat anti-rabbit (1:50,0000 dilution; ThermoFisher, 31460). Secondary antibody: Goat anti-rabbit AlexaFluor 647 (1:50,000; Invitrogen, A32733).
Validation	Anti-FtsI has been published (Wissel MC, Weiss DS. Genetic analysis of the cell division protein FtsI (PBP3): amino acid substitutions that impair septal localization of FtsI and recruitment of FtsN. J Bacteriol. 2004 Jan;186(2):490-502. doi: 10.1128/JB.186.2.490-502.2004. PMID: 14702319; PMCID: PMC305773.)
